# A new approach for improving dynamic fault ride through capability of gridctied based wind turbines

**DOI:** 10.1038/s41598-025-89396-0

**Published:** 2025-02-20

**Authors:** Shoaib Ahmed Dayo, Ahsanullah Memon, Zeeshan Anjum Memon, Touqeer Ahmed Jumani, Ghulam Abbas, Salwa Othmen, Amr Yousef, Andika Aji Wijaya

**Affiliations:** 1 Dipartimento di Industriale Ingegneria, Università degli Studi di Salerno, Via Giovanni Paolo II, 132, Fisciano, 84084 SA Italy; 2 Department of Electrical Engineering, Mehran UET, SZAB Campus Khairpur Mirs 66020, Sindh, Pakistan; 3 Department of Electrical Engineering & Computer Science, College of Engineering, A’Sharqiyah University, Ibra, 400 Oman; 4 School of Electrical Engineering, Southeast University, Nanjing, 210096 China; 5 Department of Computers and Information Technologies, College of Sciences and Arts Turaif, Northern Border University, Arar, 73222 Saudi Arabia; 6 Department of Electrical Engineering, College of Engineering, University of Business and Technology, Jeddah, 23411 Saudi Arabia; 7 Engineering Mathematics Department, Faculty of Engineering, Alexandria University, Alexandria, 21544 Egypt; 8 Mechanical Engineering Department, College of Engineering, University of Business and Technology, Jeddah, 23411 Saudi Arabia

**Keywords:** Fault ride, Doubly-fed induction generator, Wind energy, Optimization, Fuzzy logic control, Power grid, Energy science and technology, Renewable energy, Engineering, Electrical and electronic engineering, Energy infrastructure, Mechanical engineering

## Abstract

The Doubly-Fed Induction Generator (DFIG) is preferred for wind turbines (WTs) due to their variable speed capability, reinforcing energy capture efficiency. Despite its advantages, researchers continually face challenges in managing the DFIG, including overshooting, rising time, and stability under fault conditions. The faults in WTs may stem from the grid or different operational disturbances. The crowbar protection mechanism is an efficient strategy to reduce fault impacts on DFIGs. However, the traditional hysteresis-based methods to detect faults and crowbar activation are prone to false triggering, and to address the challenges posed, this paper presents a novel control strategy that increases the low-voltage ride-through (LVRT) capability of the grid-connected DFIG systems by incorporating Fuzzy Logic Control (FLC) to enhance accuracy in fault detection and employs the Salp Swarm Optimization Algorithm (SSA) to refine controller parameters. The SSA algorithm shows a superior dynamic response and stabilizes the DFIG system efficiently. Besides, the SSA algorithm precisely calibrates the proportional-integral (PI) controller gains and DC-link capacitance values to achieve the optimal transient response between Distributed Generation (DG) integration and fluctuating loads. It is evident by the results that the power quality is improved, and the active power overshoot value is decreased from 10.12 × 10^6^ to 3.78 × 10^6^. Moreover, by implementing the SSA algorithm in which the overshoot value is also decreased from 15.01 × 10^6^ to 6.10 × 10^6^, the best results are achieved. The proposed method is validated by comparative analyses with recent studies that showcase its superiority in refining machine dynamics and decreasing overshoots and transients. Henceforth, the obtained results validate the proposed method’s ability to compete against other conventional methods.

## Introduction

Globally, the quest for clean energy solutions has brought wind energy to the forefront. Among the technologies used for electricity generation from wind, the Doubly-Fed Induction Generator (DFIG) is favored within the domain of wind energy conversion for its myriad advantages over traditional fixed-speed induction generators and synchronous generators that employ comprehensive converters^1^. The DFIG stands out due to its operational flexibility across all four quadrants, permitting variable speed and bidirectional power flows while offering cost-effective conversion and reduced energy dissipation. In addition, Induction generators are typically categorized by their operational speed into two groups: those that support variable speed functionality and those that operate at a constant speed, as documented in existing scholarly texts^2^. Within these two categories, the DFIG variable speed induction generator machine is mostly used^3^. For the compact power electronic converter requirements, these generators can generate sub-synchronous and super-synchronous regions of rotor speed^4^. Integrating these machines into Wind Energy Conversion Systems (WECS) ensures both adaptability and dependability and high-quality power output.

Moreover, DFIG machines exhibit high sensitivity to both symmetrical and asymmetrical grid disturbances. Critical components such as the Rotor Side Converter (RSC) and Grid Side Converter (GSC) are at risk of damage due to various rotor winding faults that can lead to phenomena that result in poor power quality^4^. Furthermore, DFIGs have difficulties, especially when major faults^5^, such as symmetrical faults, arise on grids that are dominated by DFIGs^7^. These faults have the potential to cause major disruptions that might impair DFIG systems’ performance and stability and even shut down the grid^8^Grid code requirements (GCRs), which prioritize LVRT capabilities, are imposed globally to satisfy the WF fault ride-through criteria. For WFs, LVRT is essential since it keeps them connected during voltage sags^9^, the most common power supply problem. WFs are also anticipated to provide reactive power assistance to facilitate voltage recovery in the event of grid voltage sags. In this sense, transmission system operators (TSOs) and power system dependability establish each nation’s unique set of LVRT grid codes. International Grid code requirements are sketched in Table 1.

An all-inclusive analysis of distributed FACTS control algorithms for power quality enhancement in utility Grid with renewable energy (RE) penetration^10^. Authors in^7^comprehensively identified, classified, analyzed, and quantified power quality problems, including directly integrating RE sources in the distribution system, and thoroughly delivered mitigation methods to overcome these problems. A technique was applied in^11^to enhance wind energy penetration levels by reducing PQ issues via a distribution static compensator (DSTATCOM) controlled by the Adaptive Linear Element-Least Mean Square (ADALINE-LMS) algorithm. The control scheme in^12^discussed the variations associated with the grid’s strength, wind speed, load currents, and DC-link voltage dynamics to estimate the switching signals for the DSTATCOM. Through the implementation of a consistent current control approach, accurate switching sequences for effective regulation, and the regulation of reactive power and nonlinear load current, the DSTATCOM system plays a critical role in harmonic management. The three main parts of the DSTATCOM VSI, coupling reactors and controllers, are very efficient in solving PQ problems. Through the VSI attached to a DC capacitor, the DSTATCOM produces a regulated AC voltage while it is operating. DSTATCOM may either inject or absorb active and reactive energy into the power system as a result of the differential voltage that is created across the coupling reactor. As a result, the system’s PQ and stability are improved. DSTATCOM modifies the current in accordance with the compensation strategy to guarantee effective and steady power system functioning by employing a hysteresis band current control method, a popular technique in power electronics for controlling current within a specified range. A proportional resonant (PR) or proportional–integral (PI) controller, each integrated with an appropriate compensation method, may also be used in a DSTATCOM control system. These controllers mostly control the compensating current that the DSTATCOM injects into the AC network. While the PI controller delivers resilience in steady-state performance, the PR controller’s intrinsic resonance tracking capabilities give it an edge when managing harmonics^13^. The study in^14^discussed the challenges of integrating wind energy into the electricity grids and shed light on the available solution methodologies. The problems focused on wind energy intermittency, reactive power support, voltage and frequency stability^15^, power quality issues, fault ride-through capability, protection, cyber security, electricity market, planning, and socio-economic and environmental challenges. The study in^16^explores the necessity for fault-ride-through capability from a power system security point of view. Authors in^17^critically assessed the current FRT standards (grid-code requirements) and FRT strategies implemented/ proposed for wind energy conversion systems (WECSs), solar photovoltaic (PV) systems, and microgrids. A microgrid powered by a 500-kilowatt (kW) solar system was developed to enhance conventional power distribution, minimize system weight, and ensure functionality during essential failure scenarios. Multi-objective optimization tasks were performed, and the system determined the optimal values for a variety of parameters, such as maximum power, Maximum Power Point Tracking (MPPT) voltage, battery terminal voltage, and panels per string^18^. The Salp Swarm Algorithm (SSA) enhances the interplay between controller configurations^19^. It details how the proportional and integral gains of the grid-side and rotor-side controllers, along with the phase-locked loop, are meticulously calibrated to optimize the parameters and dynamic behavior of systems using an SSA-based framework^20^.

Building on the DFIG optimization work, researchers have also focused on enhancing machine dynamics of related technologies like PMSGs and BDFIGs. Researchers have demonstrated the variable speed PMSG optimization method to enhance machine dynamics^21^controllers, which are optimized through the Whale Optimization Algorithm, to regulate the current directed through the machine side converter (MSC)^6^. Prior research was dedicated to advancing the fault tolerance, fast transient reaction, and steady-state performance of permanent magnet synchronous generators (PMSGs) since continuous energy generation and satisfying grid regulations are imperative^22^. Many researchers worldwide have used a variety of soft-computational techniques, including particle swarm optimization (PSO), the General Regression Neural Network (GRNN) algorithm, and an enhanced version of PSO, to study the power flow control of a grid-connected MG. The main goal of all the studies was to get ideal power regulation without using the laborious and ineffective traditional PI tuning methods. According to the findings of our investigations, the PI coefficients chosen using the aforementioned soft computational optimization approaches have improved the transient behavior of the grid-connected MG systems under consideration when compared to traditional PI tuning methods^19,23^.

There are some significant drawbacks to the aforementioned optimization techniques (GA and PSO). For example, the GA is not suitable for working with dynamic data sets and can become locked in the local solution. Due to these drawbacks, GA is no longer a viable optimization method for the newest MG controls. However, when dealing with high-dimensional optimization issues, PSO typically becomes stuck in the local minimum (local solution). Additionally, it has drawbacks such as a sluggish rate of convergence and ambiguity in parameter selection. Although PSO’s searching ability is rather strong in the early versions, it struggles to find the best answer for several benchmark functions^24^.

Various computational optimization approaches, including the Gray Wolf Optimizer (GWO), Particle Swarm Optimizer (PSO), and Whale Optimizer Algorithm (WOA), have been introduced in past work to improve the transient responsiveness and smooth operation of PMSG systems. Compared to the conventional PI controller, PSO demonstrates better performance in limiting fault current (If). Both GWO and WOA have improved convergence properties, as demonstrated by Zhang et al. and Mahmoud et al.^22,25^.

The Salp Swarm Algorithm (SSA) has been utilized in prior studies to enhance the dynamic response and low-voltage ride-through capability of doubly-fed induction generators^3^. This advanced optimization technique boosts transient performance. It emphasized the efficiency of the proposed control method when set against a controller that was justified in PSO and assessed under identical operational scenarios. Limiting fault current, controlling active/reactive power, and keeping transient stability are the primary points of comparison^26^. In the above research, the authors used different techniques, but the SSA optimization technique with a DFIG machine was found to be the best way to improve machine dynamics.

Recent technological advancements have paved the way for rapid fault detection methodologies, as reported in contemporary research. A modern machine learning model for the early detection of Inter-Turn Short-Circuit faults within stator windings of doubly-fed induction generators has been introduced, utilizing real-time current data^27^. Additionally, a Fuzzy Logic Controller (FLC) design incorporates voltage magnitude and frequency measurements from power windings to determine low-voltage ride-through conditions^22^. The FLC has improved problem detection and converges faster, while the analytical model resulted in a lowering power winding voltage drop. Moreover, in Ref^15^. , the authors designed a brake chopper system based on a hysteresis controller. The system shows enhanced FRT performance and maintains the DC-link voltage within safe levels. However, the Voltage deviation method VDM does not rely on communication. All or some converters take part in controlling the DC voltage and appropriately sharing the power imbalance at the same time in VDM, which is derived from power-frequency droop control in AC systems^15^. Compared to master-slave control, VDM is more reliable and does not cause voltage oscillation like the voltage margin method VMM does. VDM has garnered a lot of study attention because of its characteristics. The DC voltage deviating from its rated value, the frequency deviation in the adjacent AC system, and power flow calculation methods incorporating AC and DC systems are just a few of the issues that arise from disturbance under fixed droop control, even with VDM’s widespread use. MTDC system responds to power disruptions, such as the loss or failure of one or more converters, the disconnection of a DC line, and typical power setting adjustments, which are specific problems. The converter’s power with fixed droop control may reach its limitations during a power outage; as a result, these converters cease to participate in power sharing, and the DC voltage divergence may be significant^28^. Mahmoud et al.^8^reported that the Fuzzy-PID (FPID) controller output variables have scaling factors optimized by genetic programming^29^. The overarching goal was to enhance system responsiveness under disturbances by integrating the superior attributes of PID and FPID controllers. A focused approach, detailed in, led to the development of a Takagi-Sugeno-Kang (TSK) fuzzy model, a pivotal aspect presented in Ref^30^. This refined model is engineered to optimize the harnessing of energy from wind turbines operating at diverse rotational speeds. A detailed summation of various DFIG strategies is articulated in Table 2.

**Table 1:** International grid code requirements.

Country	During fault condition		After fault condition	
	$\:{\varvec{V}}_{\varvec{m}\varvec{i}\varvec{n}}$ (pu)	$\:{\varvec{T}}_{\varvec{m}\varvec{i}\varvec{n}}$ (s)	$\:{\:\:\:\:\:\:\:\varvec{V}}_{\varvec{m}\varvec{i}\varvec{n}}$ (pu)	$\:{\varvec{T}}_{\varvec{m}\varvec{i}\varvec{n}}$ (s)
**Australia**	0.00	0.1	0.7	2
**China**	0.16	0.625	0.9	3
**Canada**	0	0.15	0.85	1
**Denmark**	0.2	0.5	0.9	1.5
**Germany**	0	0.15	0.9	1.5
**India**	0.15	0.3	0.85	3
**Ireland**	0.15	0.625	0.9	3
**New Zealand**	0	0.2	0.6	1
**United States**	0.15	0.14	0.8	0.21
**United Kingdom**	0.15	0.625	0.9	3

**Table 2:**
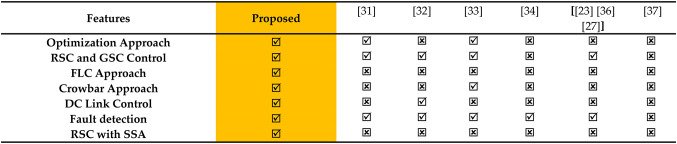
Existing summary of DFIG control techniques.

However, authors in^38^ have applied a modified demagnetization control strategy to improve the LVRT for DFIG. It is expected that the optimization technique and Fuzzy Logic controller will produce more satisfactory results along with the above-mentioned control strategy in the future.

The Comprehensive review of the literature indicated that the main focus of the researchers was on improving the dynamic response of DFIG with certain algorithms and controllers. However, the existing controllers and algorithms have certain practical limitations. As a result, the selection of proportional and integral gain is not optimal, and the solution cannot converge to an optimal value, jeopardizing the dynamic response of DFIG. This research diverges from preceding studies by applying the Salp Swarm Algorithm (SSA) for enhanced machine dynamic optimization and implementing a Fuzzy Logic Controller (FLC) for streamlined fault detection. The following are the key contributions of this manuscript:

- The DFIG model is developed with RSC to control the active/reactive power during initial fault conditions.
- An optimization algorithm, SSA, is employed in RSC to improve the machine dynamics at the time of initial conditions.
- A fuzzy logic controller is implemented to detect the occurrence of the fault.
- The results obtained are compared with those of the PSO and hysteresis detection methods.
- Finally, the superiority of the proposed work is compared with the latest published literature.

The main contribution of this paper is to improve the transient conditions for DFIG during LVRT conditions using the SSA optimization technique. Moreover, fuzzy logic controllers have been used earlier to detect faults to avoid an increase in current that may damage the converters.

### Low voltage ride through (LVRT) capability characteristics of the proposed work

The control strategy proposed in this study has been designed to enhance the Low Voltage ride-through (LVRT) capability of grid-tied wind turbines, ensuring that they remain operational and contribute to grid stability during fault conditions. To evaluate its performance, we conducted simulations under various voltage sag scenarios, with voltage levels ranging from **0.2 pu to 0.9 pu**. The key aspects of the system’s performance are:

#### Rotor Current Limitation

During faults, one of the primary concerns is the rise in rotor current, which can damage the rotor-side converter if not managed properly. The proposed control strategy includes a **current saturation mechanism** that limits rotor currents to a maximum of **2.0 p.u**, effectively protecting the converter and ensuring system safety.

#### Reactive power (Q) injection

Reactive power support during voltage sags is essential to stabilize the grid and aid in voltage recovery. The control system actively adjusts to inject **reactive power** during fault conditions, meeting the requirements of international grid codes, such as **IEC 61400-21**, which demand grid support from wind turbines during low voltage scenarios.

.

## The proposed methodology

**Fig. 1:**
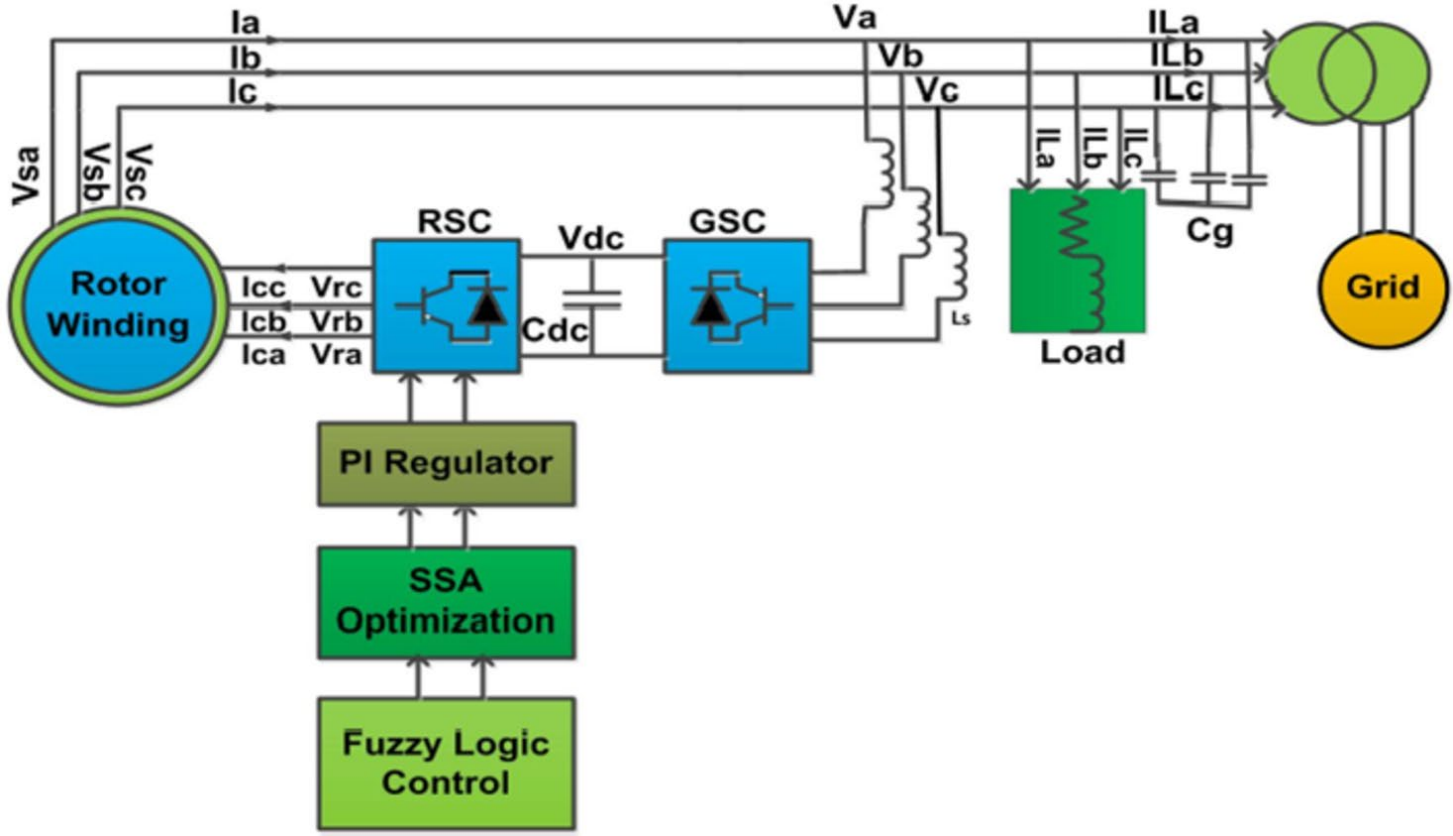
Schematic of the Proposed Model.

This section introduces the proposed DFIG model and control methodology to improve fault ride-through capability. The DFIG differs from a directly grid-connected synchronous generator in its connection methodology. In the DFIG, the stator is directly integrated with the grid, and the rotor is connected through a back-to-back power converter, as illustrated in Fig. 1. This bidirectional converter comprises two key components: the Grid Side Converter (GSC) and the Rotor Side Converter (RSC). The GSC’s role is to manage the DC link voltage. In contrast, the RSC focuses on controlling reactive and active power generation by manipulating the rotor current. Specifically, the RSC adjusts the current components of the excitation injected into the rotor circuit, modifying the DFIG system’s reactive and active power outputs.

In a doubly-fed induction generator (DFIG), the stator directly connects to the grid, while brushes and slip rings link the rotor to the grid. Cascaded bidirectional converters consist of a grid side converter (GSC), a rotor side converter (RSC), and a DC link capacitor connected between the converters^6^. The capacitor is sized to supply a DC link voltage equal to twice the stator voltage To protect the converters from overvoltage damage^39^. In both super-synchronous and sub-synchronous modes, the converter transfers energy bi-directionally. While the GSC regulates the DC link voltage, the RSC modulates the rotor current injection to control active/ reactive power output^4^.

Currently, many researchers are working on DFIG modeling, as presented in^40^. However, the existing models have certain limitations. The DFIG system was simulated using the MATLAB / Simulink toolkit, set within a synchronously rotating d-q reference frame. The ‘Park model’ fifth-order induction generator was simulated. In this setup, the stator flux aligns with the d-axis directly, and the reference frame’s rotation speed matches the stator voltage’s rotation speed. As far as the current limitation during LVRT is 2.0 p.u. exceeding the above value may damage the converters, so the current is limited under this value. At last, rotor current and electrical torque can be independently controlled.

Transient analysis of an induction generator is performed using the equivalent diagram depicted in Fig. 1, which is derived from stator and rotor fluxes integrated with the grid and is based on the following set of mathematical equations^41^.

The model applies standard dq coordinate transformations to represent the DFIG voltages, fluxes, and other parameters as detailed in Eqs. (1)-(7). This approach allows independent control of the rotor current and torque.

For a dq reference frame grid-aligned induction machine, the equations for the stator, rotor voltage, and flux are presented in^42^.

1 $$\:\overrightarrow{{V}_{s}}={R}_{s}\overrightarrow{{I}_{s}}+\frac{d\overrightarrow{{\phi\:}_{s}}}{dt}+{\omega\:}_{s}\overrightarrow{{\phi\:}_{s}}$$

2 $$\:\overrightarrow{{V}_{r}}={R}_{r}\overrightarrow{{I}_{r}}+\frac{d\overrightarrow{{\phi\:}_{r}}}{dt}+j{\omega\:}_{sl}\overrightarrow{{\phi\:}_{r}}$$

3 $$\:\overrightarrow{{\phi\:}_{s}}={L}_{s}\overrightarrow{{I}_{s}}+{L}_{m}{\overrightarrow{{I}_{r}}}_{\:};{{}_{\:}{}^{\:}\phi\:}_{r}={L}_{m}\overrightarrow{{I}_{s}}+{L}_{r}\overrightarrow{{I}_{r}}$$

4 $$\:\overrightarrow{{V}_{s}}={R}_{s}\overrightarrow{{I}_{s}}+\frac{d\overrightarrow{{\phi\:}_{s}}}{dt}+j{\omega\:}_{s}\overrightarrow{{\phi\:}_{s}}$$

5 $$\:\overrightarrow{{V}_{r}}={R}_{r}\overrightarrow{{I}_{r}}+j\left({\omega\:}_{s}-{\omega\:}_{r}\right)\overrightarrow{{\psi\:}_{r}}+\frac{d\overrightarrow{{\psi\:}_{r}}}{dt}$$

6 $$\:\overrightarrow{{\phi\:}_{s}}={L}_{s}\overrightarrow{{I}_{s}}+{L}_{r}\overrightarrow{{I}_{r}}$$

7 $$\:\overrightarrow{{\phi\:}_{r}}={L}_{m}\overrightarrow{{I}_{s}}+{L}_{r}\overrightarrow{{I}_{r}}$$

8 $$\:{L}_{s}={L}_{s\sigma\:}+{L}_{m}$$

9 $$\:{L}_{r}={L}_{r\sigma\:}+{L}_{m}$$

The rotor and stator windings in the generator model are characterized by voltages and fluxes, denoted as $\:\overrightarrow{{V}_{r}}$ ​, $\:\overrightarrow{{V}_{s}}$, $\:\overrightarrow{{\phi\:}_{r}}$ and $\:\overrightarrow{{\phi\:}_{s}}$ respectively. This model incorporates rotor and stator resistances, labeled as *Rr*​ and *Rs*​, along with mutual inductance *Lm​* and leakage inductances $\:{L}_{r\sigma\:}$. The rotor’s electrical angular velocity is represented as ωr​. A leakage factor σ is also defined as shown below, where σ represents the ratio of leakage inductance to total inductance for each winding:.

10 $$\:\sigma\:=1-\frac{{L}_{m}^{2}}{{L}_{s}\times\:{L}_{r}}$$

From Eqs. (6) and (7), the stator and rotor current expression can be represented as the function of fluxes;

11 $$\:\overrightarrow{{I}_{s}}=\frac{1}{{L}_{s}-\left(\frac{{L}_{m}^{2}}{{L}_{r}}\right)\overrightarrow{{\phi\:}_{s}}}-\left(\frac{{L}_{m}}{{L}_{r}}\right)\frac{1}{{L}_{s}-\left(\frac{{L}_{m}^{2}}{{L}_{r}}\right)\overrightarrow{{\phi\:}_{s}}}$$

12 $$\:\overrightarrow{{I}_{r}}=-\left(\frac{{L}_{m}}{{L}_{r}}\right)\frac{1}{{L}_{r}-\left(\frac{{L}_{m}^{2}}{{L}_{s}}\right)\overrightarrow{{\phi\:}_{s}}}+\frac{1}{{L}_{r}-\left(\frac{{L}_{m}^{2}}{{L}_{s}}\right)\overrightarrow{{\phi\:}_{r}}}$$

The Eqs. (11) and (12) can further be modified to simpler forms as presented below,

13 $$\:\overrightarrow{{I}_{s}}=\frac{1}{A}(\overrightarrow{{\phi\:}_{s}}-{k}_{r}\overrightarrow{{\phi\:}_{r}})$$

14 $$\:\overrightarrow{{I}_{r}}=-\frac{1}{B}({k}_{s}\overrightarrow{{\phi\:}_{s}}-\overrightarrow{{\phi\:}_{r}})$$

where $\:{k}_{s}$, $\:{k}_{r}$ are constant values that symbolize the coupling factors for the stator and rotor, respectively. These are mathematically presented as shown.

15 $$\:{k}_{s}=\frac{{L}_{m}}{{L}_{s}};{k}_{r}=\frac{{L}_{m}}{{L}_{r}}$$

Similarly, A and B in Eqs. (13) and (14) can be represented as;

16 $$\:A={L}_{s}-\frac{{L}_{m}^{2}}{{L}_{r}}$$

17 $$\:B={L}_{r}-\frac{{L}_{m}^{2}}{{L}_{s}}$$

These parameters correspond to the transient stator inductance (*Ls*′​) and transient rotor inductance (*Lr*′​) in a synchronous machine, respectively, as represented by:

18 $$\:L{\prime }_{s}={L}_{s\sigma\:}+\frac{{L}_{r\sigma\:}{L}_{m}}{{L}_{r\sigma\:}+\:{L}_{m}}$$

19 $$\:L{\prime }_{r}={L}_{r\sigma\:}+\frac{{L}_{s\sigma\:}{L}_{m}}{{L}_{s\sigma\:}+\:{L}_{m}}$$

**Fig. 2:**
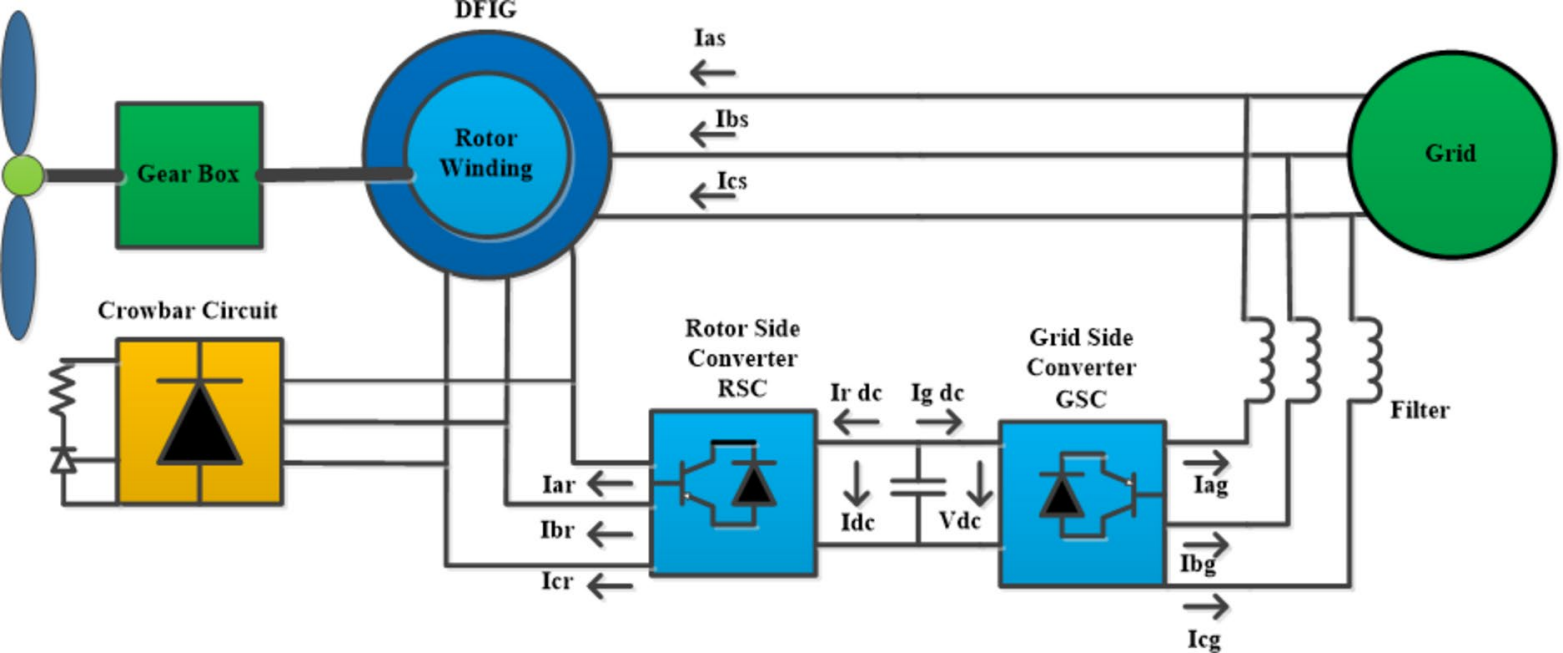
Graphical representation of double-fed induction generator (DFIG).

It can also be described using the equivalent circuit diagrams depicted in Fig. 2. The mathematical representation of the peak short-circuit current produced by the DFIG in response to a fault is detailed in Eqs. (4)–(18). Differential voltages delineated in Eqs. (4) and (5) serve to determine the steady-state current. However, an integrated solution for the DFIG is obtained by combining the resolution of the homogeneous differential Equation with the steady-state current solution.

Free currents ensure electrical energy flow continuously by switching from one stationary state to another when a fault occurs. Like a synchronous generator^4^, the DFIG “free currents” rotate at almost synchronous speed. The generator produces three distinct current components at different frequencies by transforming currents from one state to another. Three types of currents are involved: the stator DC current, rotor DC current, and stationary currents^42^.

## The proposed optimization algorithm

This paper leverages a Salp Swarm Algorithm (SSA) to optimize the DFIG’s proportional-integral (PI) control gains by addressing the limitations of the existing methods. SSA is the contemporary algorithm for solving different optimization problems. It has been applied to find better results than other optimization algorithms in solving various engineering problems, like load frequency control. This section presents the mechanism and implementation of SSA optimization in detail. The SSA was selected as the optimization technique due to its transparent operational process, proficiency in circumventing local minima, and adeptness at maintaining equilibrium between explorative and exploitative tactics. The function applied for optimizing the PI gains is detailed in Eq. 20.

20 $$\:FF={\int\:}_{0}^{t}t\left|{e}_{1}\right(t\left)\right|dt+{\int\:}_{0}^{t}t\left|{e}_{2}\right(t\left)\right|dt+{\int\:}_{0}^{t}t\left|{e}_{3}\right(t\left)\right|dt+{\int\:}_{0}^{t}t\left|{e}_{4}\right(t\left)\right|dt+{\int\:}_{0}^{t}t\left|{e}_{5}\right(t\left)\right|dt+{\int\:}_{0}^{t}t\left|{e}_{6}\right(t\left)\right|dt$$

In this context, ‘t’ denotes the elapsed simulation time, and the magnitudes |e1(t)| to |e6(t)| correspond to the error signals for their respective control loops. The subsequent section presents the workings of the SSA and its integration within the current study.

### SSA optimization algorithm

Basically, the salps belong to the famous family of Salpidae group with a translucent barrel-shaped body. Mathematically, to model its behavior movement, the initial positions are initialized randomly, as portrayed in Eq. (21)^3^.

21 $$\:{k}_{1}^{1:n}=\text{ran}(.).\text{*}\left(\mu\:{b}_{j}-l{b}_{j}\right)+l{b}_{j},\forall\:j\in\:{\rm no.of\:variables}$$

The variables $\:{k}_{1}^{1:n}$ signify the initial positions of the salps, µbj​, and lbj​ denote the upper and lower bounds, respectively, and “ran” indicates random numbers ranging from 0 to 1. Following the stochastic dispersal of the search agents, the salp swarm is divided into ‘leaders’ and ‘followers.’ The first half of the swarm, distinguished by better solution quality, assumes the role of leaders, with the remainder classified as followers. The evaluation of the fitness function for the leading salps is updated and recorded at the end of each iteration, as specified in Eq. (21).

22 $$\:{k}_{j}^{1}={M}_{i}+{c}_{1}\left(\right(\mu\:{b}_{j}-l{b}_{j}){c}_{2}+l{b}_{j});{c}_{3}\ge\:0.5$$

23 $$\:{M}_{i}={c}_{1}\left(\right(\mu\:{b}_{j}-l{b}_{j}){c}_{2}+l{b}_{j});{c}_{3}<0.5$$

Where c1, c2, and c3 are random values, and the symbol denotes the leader salp location in the ith and the jth dimensions of food supply, respectively. Due to its adaptive optimization, SSA rarely bypasses local minima compared to conventional optimization techniques such as GA and PSO. The SSA updates the location of the trailing salps, and the trailing salps gradually begin to move toward the leading salps. This aids the SSA in avoiding the trap of becoming stuck at local optima. As a result, SSA generates a solution that is either optimal or very near to optimal solution. The capacity to balance exploration and exploitation is another area in which SSA excels. The leader Salp’s position update is determined exclusively by the proximity to the food source, a process quantified in Eq. (22). The coefficient c1​ plays a crucial role within the SSA framework, facilitating a balance between the exploration and exploitation phases, with its value derived mathematically as presented in Eq. (23).

24 $$\:{c}_{1}=2{e}^{-{\left(\frac{4 L}{L}\right)}^{2}}$$

Here, ‘L’ represents the maximum iteration count, and ‘I’ signifies the current iteration. Furthermore, the updated position of the salp follower is calculated by applying Newton’s second law of motion, as described in the following section;

25 $$\:{k}_{j}^{i}=\frac{1}{2}\times\:a{t}^{2}+{v}_{0}t$$

$\:\:\:\text{w}\text{h}\text{e}\text{r}\text{e}{k}_{j}^{i}$ Symbolises the ith position in slap follower in jth dimension and i$\:\ge\:$2, t denotes time and $\:{v}_{o}\:$shows the slap velocity at the initial stage of the optimization process and is generally considered as 0. Since the iteration count takes the place of time during the optimization process and to prevent fractional numbers between consecutive iterations, Eq. (25) can be reformulated as described below.

26 $$\:{k}_{j}^{i}=\frac{{k}_{j}^{i}-{k}_{j}^{i-1}}{2}$$

where, $\:{k}_{j}^{i}$ and i$\:\ge\:2$ shows the ith position of slap follower in the jth dimension.

### Implementation of SSA in the proposed study

This study proposes a methodology that employs the Salp Swarm Algorithm (SSA) for deriving optimal Proportional-Integral (PI) parameters for the Rotor-Side Converter (RSC) to enhance the dynamic response of the grid-tied DFIG system,

In the presented study, three distinct optimization techniques are utilized—Internal Model Control (IMC), Particle Swarm Optimization (PSO), and Salp Swarm Optimization (SSA)—aimed at optimizing the dynamic performance of the DFIG within wind energy conversion systems. The IMC method focuses on deriving PI controller parameters that assure a desired closed-loop bandwidth and time constant, adjusting the system’s open-loop poles alongside the PI controller’s zeros considering the system’s operational parameters^41^. On the other hand, swarm intelligence techniques such as PSO and SSA^43^ operate based on iterative optimization, eliminating the need for a comprehensive mathematical model of the system. These techniques initiate the optimization process with a randomized spread within a predetermined search space, progressively assessing and modifying the positions of search agents according to their performance, continuing this iterative process up to a pre-established number of iterations, as outlined in Eqs. (20) and (21).

SSA is mainly implemented in these applications due to their simplicity, flexibility, and effectiveness in handling complex, multi-modal optimization landscapes that are typical in dynamic system settings like DFIGs. It efficiently finds optimal solutions that traditional gradient-based optimization methods might miss due to their susceptibility to local minima. By implementing SSA, researchers can significantly enhance the operational efficiency and resilience of DFIG systems, which are crucial for integrating wind energy smoothly and reliably into the power grid.

The simulation model for the DFIG and the associated control methodology is constructed within the Simulink environment, while the programming for the SSA is performed in MATLAB’s R2016b script editor. The SSA algorithm’s preparatory phase involves setting up optimization parameters such as iteration count, number of search agents, their limits, decision variables, and the simulation time frame. The objective is to pinpoint the optimal PI controller gains by minimizing an error-centric fitness function. The SSA follows a metaheuristic approach, initiating its search by scattering a set number of agents within a specified search domain. Subsequently, it calculates and orders the fitness of each agent, using their performance to guide the search. The fitness values are continually assessed against the best results from previous iterations. When a new solution surpasses the fitness of earlier solutions, it is adopted as the new global optimum; otherwise, the algorithm retains the existing superior solution.

The pseudo-code on the SSA is depicted below:


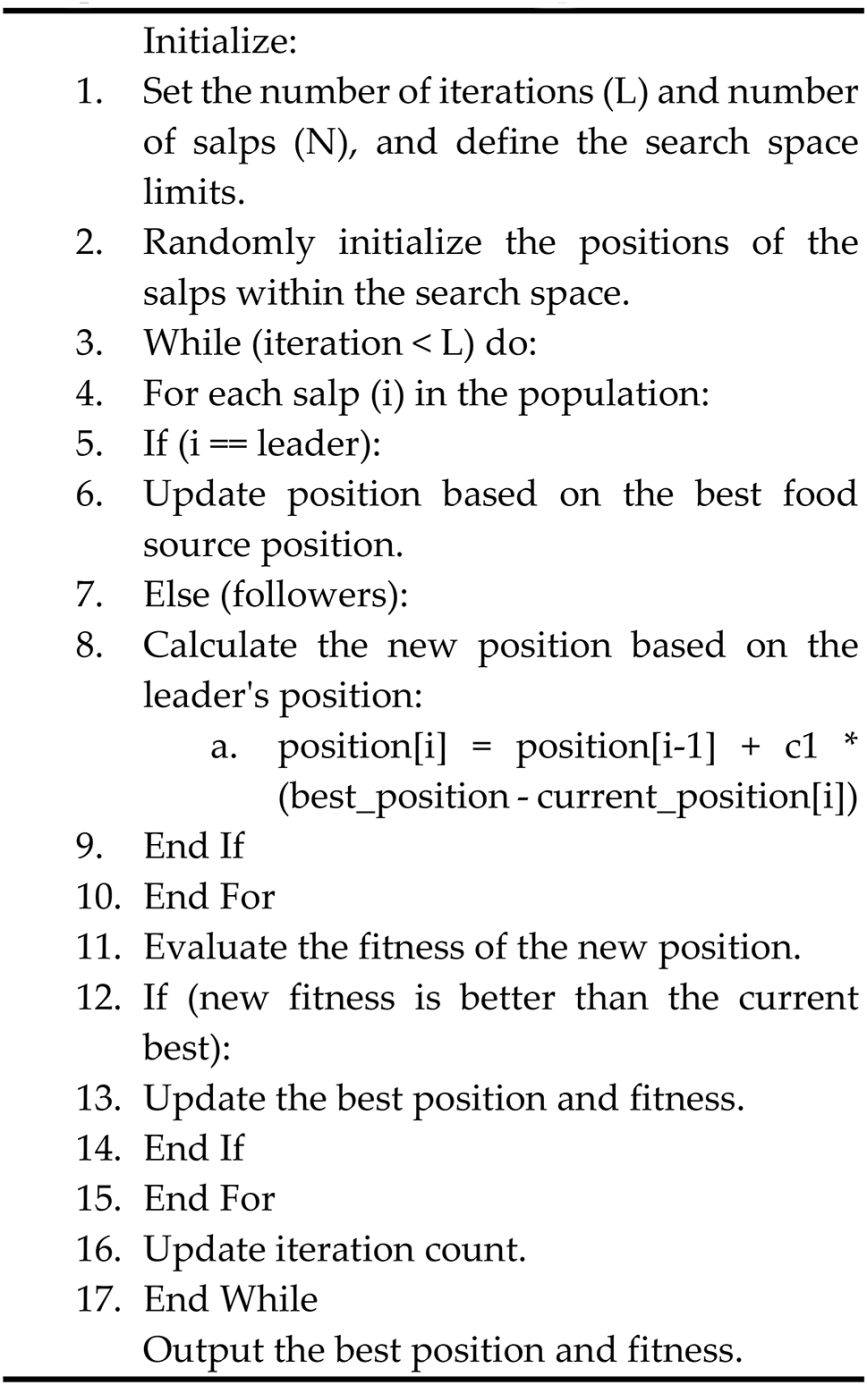


Upon completion of the simulation and the successful minimization of the fitness function, the PI controller gains for both the RSC and GSC are optimized. The efficacy of the proposed approach is substantiated through a comparative analysis of the dynamic responses under normal and faulty conditions against those controlled by the PSO in a standardized system setup and identical operating scenarios. The specific parameters for both SSA and PSO optimizations are detailed in Table 3.

## Fuzzy logic Controller

A Fuzzy Logic Controller (FLC) is implemented to swiftly and accurately detect faults based on voltage^30^and frequency measurements^44^. The design of the FLC involves three critical stages: fuzzification, establishing control rules within the fuzzy logic engine, and applying the inference engine.

### Fuzzification

This study proposes a Fuzzy Logic Controller (FLC) which uses two inputs, i.e., instantaneous voltage. $\:{V}_{m}$ and frequency $\:{F}_{m}$. For these two parameters, the discussion ranges are [0, 1] and^6^.

### Knowledge base

In the knowledge base configuration crucial to constructing the proposed fuzzy logic controller, the controller is architected to discern and respond aptly to specific grid conditions.

1. When there is a high voltage drop, and the frequency is not unaltered, the FLC must turn on the compensation mode of RSC.
2. When there is a high voltage drop, and the frequency is unaltered, the FLC must turn on the RSC Compensation mode.
3. When there is no change in frequency and voltage drop, the RSC must work in normal operation mode.
4. When there is a high change in frequency with no voltage drop, the RSC Compensation mode must be turned on.

The fuzzy logic controller produces a binary output, activating the rotor side converter’s compensation mode during faults, ensuring consistent operation during system stability. This membership function’s output, denoted as (Ir), operates using four linguistic variables: zero (ZO), positive small (PS), positive medium (PM), and positive big (PB), each contributing to defining the controller’s operational responsiveness and adaptability. The proposed study obtains the rules based on Table 2 using a similar approach.

### Inference engine

The operational essence of the fuzzy logic controller is the integration of predefined rules to generate an appropriate control action. In this study, we utilize the individual rule firing mechanism that is standard in fuzzy logic inference, applying the Mamdani method^20^. The inference engine activates rules individually, determining the control action based on the least degree of membership amongst the inputs. The process of individual rule activation using the Mamdani inference method^31^ and the min-inference technique is demonstrated in the following instances:

1. When $\:{V}_{m}$registers as PS with a membership value of 0.76747 and $\:{F}_{m}\:$is ZO with a membership value of 1.0, $\:{I}_{r}$ is assigned as PS with a membership value equal to the minimum of the two, which is 0.76747.
2. When $\:{V}_{m}$ is denoted as PM with a membership value of 0.1550 and $\:{F}_{m}$ is ZO with a membership value of 1.0, $\:{I}_{r}$ is classified as PM, with a membership value derived from the minimum of the two, which is 0.1550.

The designed fuzzy logic controller is tasked with outputting a binary signal. This output governs the activation of the rotor side converter’s compensation mode during faults and ensures regular operation in stable conditions. The output, denoted by $\:{I}_{r}$, is the singular output from the proposed fuzzy logic controller and is characterized by four linguistic descriptors: zero.

**Table 3:** The proposed fuzzy logic controller is based on rules.

Rule #	$\:{V}_{m}$	$\:{F}_{m}$	$\:{I}_{r}$	Rule #	$\:{V}_{m}$	$\:{F}_{m}$	$\:{I}_{r}$
01	PS	PS	PS	09	PS	PM	NM
02	ZO	PS	ZO	10	ZO	PM	NS
03	PM	PS	PM	11	PM	PM	PS
04	PB	PS	PB	12	PB	PM	ZO
05	PS	ZO	NS	13	PS	PB	NB
06	ZO	ZO	PS	14	ZO	PB	NM
07	PM	ZO	ZO	15	PM	PB	NS
08	PB	ZO	PM	16	PB	PB	PS

(ZO), positive small (PS), positive medium (PM), and positive big (PB).

## Results and discussion

**Fig. 3:**
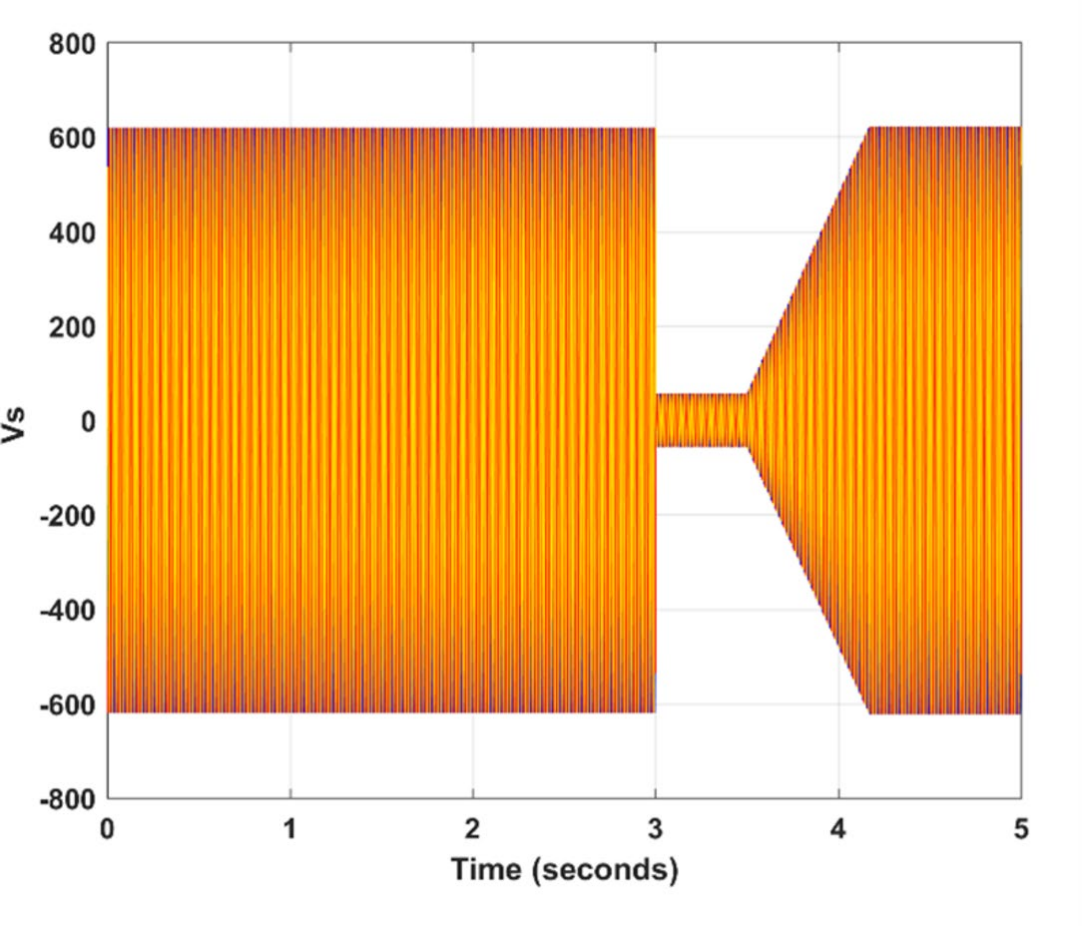
Performance of proposed model’s stator voltage (Vs) along with defined time span.

**Fig. 4:**
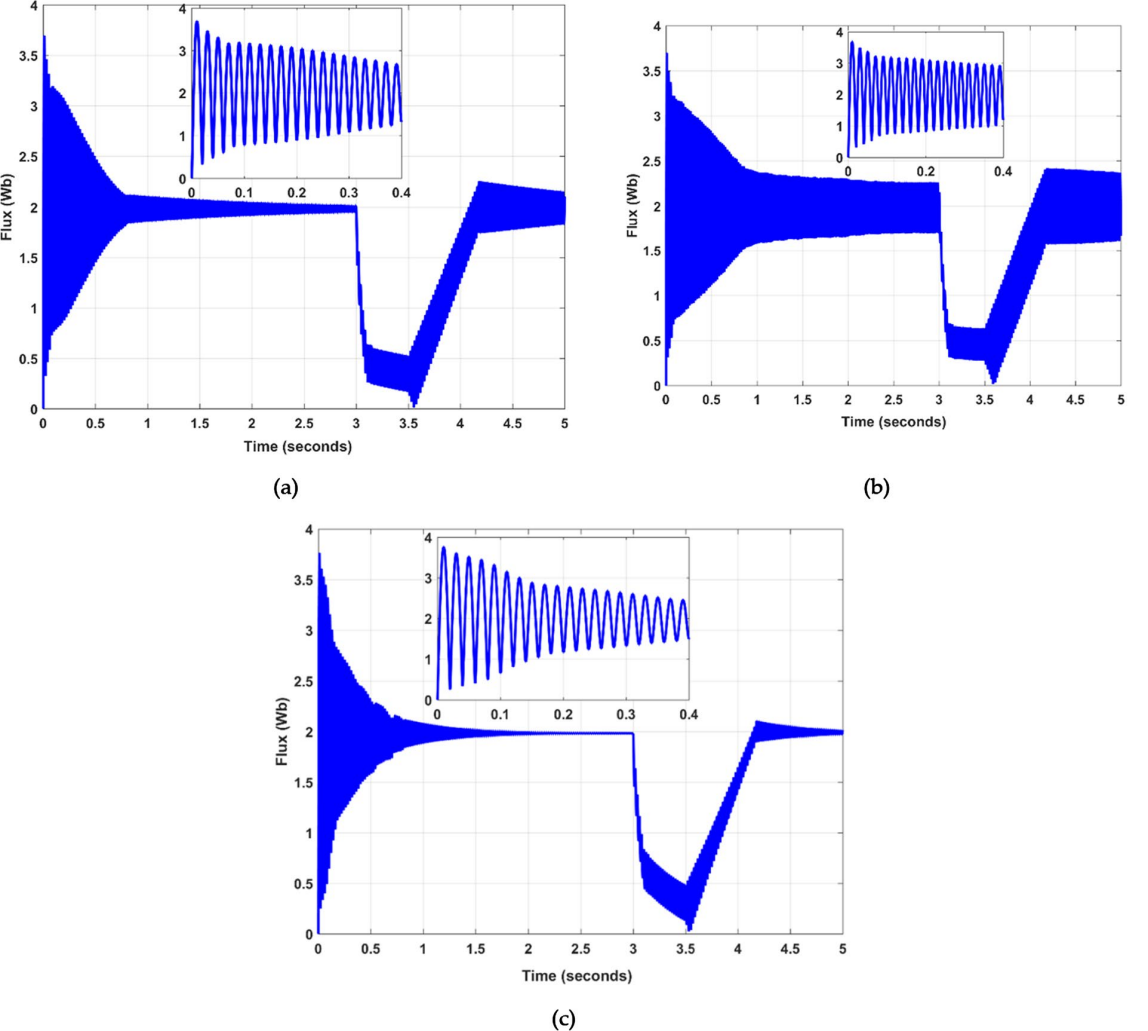
Flux performances of different scenarios over a specified time, (**a**) with implemented SSA, (**b**) without implemented PSO and SSA, and (**c**) PSO implemented only.

The simulation parameters and other relevant values are given in Table 4. The results demonstrate that the proposed SSA optimization and FLC fault detection techniques substantially improve LVRT capability and dynamic response compared to standard and PSO-based approaches. Specific benefits include reduced active and reactive power overshoots, limited fault currents, and smooth flux regulation. This section presents the performance outcomes of the controller to ascertain its efficacy. It includes a comparative analysis of the SSA-based controller against one optimized using the PSO technique. The simulations were conducted using MATLAB / SIMULINK, where a symmetrical short-circuit fault was introduced at 3 ms and subsequently cleared at 4.17 ms. In the tested back-to-back voltage source converter configuration, the stator receives its three-phase supply voltage and is directly linked to the grid. Conversely, the rotor is connected via a Rotor-Side Converter (RSC) and a Grid-Side Converter (GSC). Figure 3 illustrates a voltage dip occurring between 3 ms and 4.17 ms; Fig. 3 illustrates a voltage dip to 75 V. The machine’s operational performance is consistent with theoretical anticipations during the simulated fault condition. Figure 4 (a), (b), and (c) demonstrate the flux characteristics under optimized and conventional methods. A notable inversion in the flux trajectory from positive to negative is observed immediately upon fault occurrence, as depicted in Fig. 4 (b). Maintaining flux is imperative for consistent machine operation in all conditions. A DFIG necessitates adequate flux levels to effectively convert the mechanical energy harvested from the wind turbine’s blades into electrical energy, and this is facilitated by the interplay between the rotor’s magnetic field and the stator, which induces voltage and current in the stator coils, allowing for meticulous control over the DFIG’s output power through strategic modulation of the rotor flux.

**Table 4:** Simulation parameters and their values.

Parameter	Values	Parameter	Values
Grid Frequency	50 Hz	Capacitor Bus	80 × 10^−3 F
Rated Stator Voltage	690 V	DFIG Rated Capacity	2.0 MVA
Rated DC link Voltage	1150 V	Grid Resistance	200 × 10^−6 Ω
Mutual Inductance	2.5 × 10^−3 H	Stator / Rotor Resistance	2.6 × 10^−3 / 2.9 × 10^−3 Ω
Grid Inductance (Lg)	400 × 10^−6 H	Rotor Moment of Inertia (J)	127 / 2

Whereas in Fig. 4 (c), the suggested controller can return the flux flow to its nominal value once a defect has occurred. The machine and connected devices are protected from overheating because the flux instantly falls in magnitude once a fault is injected, lowering an EMF and the current. When compared to Fig. 4 (a) and (c), the results demonstrate that the calculated result is excellent.

### Active power (P) and reactive power (Q) relationship

**Fig. 5:**
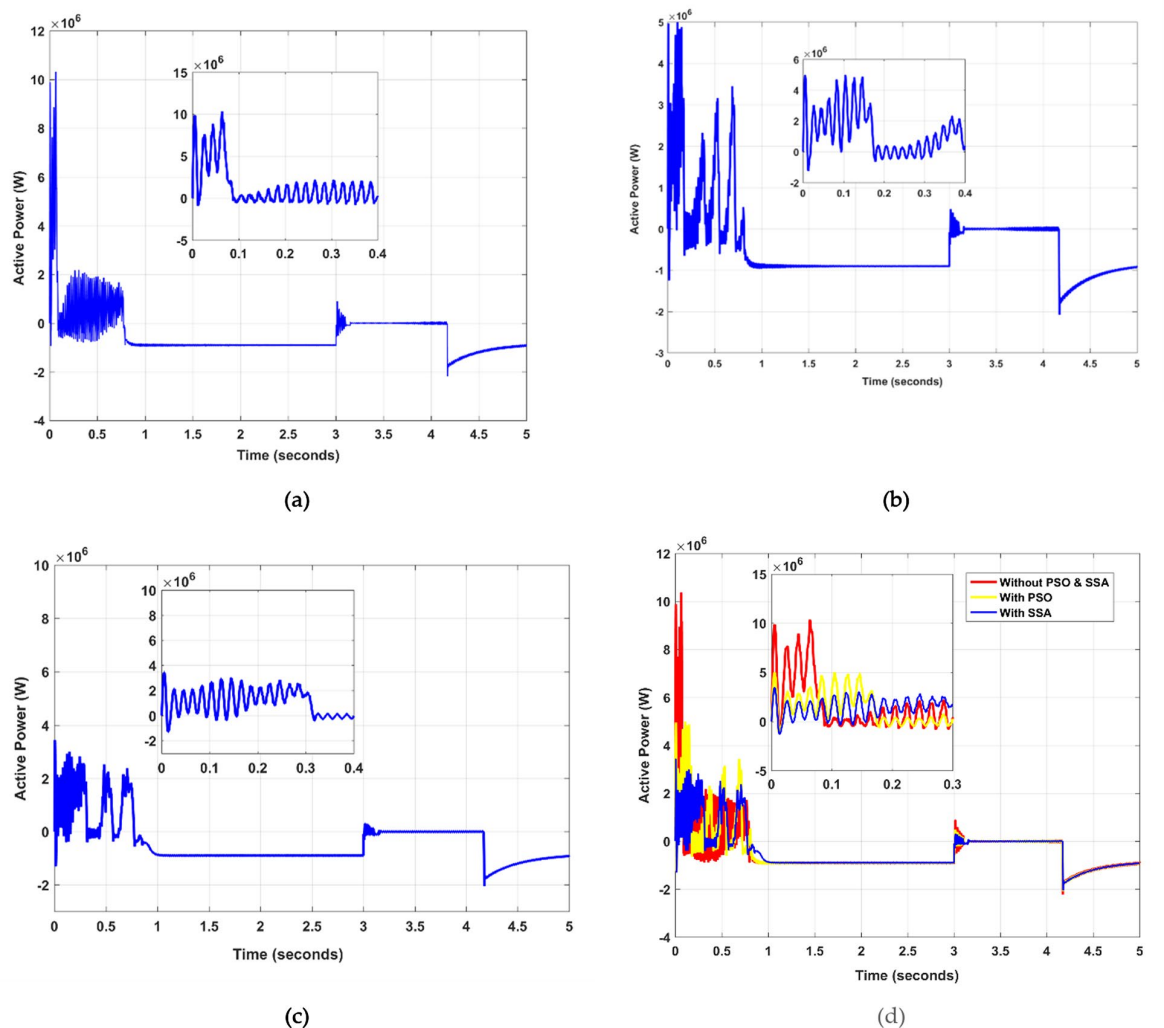
Active Power (P) performance with defined time, (**a**) without PSO and SSA, (**b**) with PSO, and (**c**) proposed with SSA (**d**) merged figures.

Regulating the active power of a DFIG during both standard and fault conditions is crucial. Observations of the DFIG-based wind turbine’s active power at startup and during faults reveal a significant initial overshoot, as presented in Fig. 5 (a). The application of the PSO algorithm, as shown in Fig. 5 (b), results in a substantially reduced initial overshoot. Implementing the SSA optimization algorithm further mitigates the active power (P) overshoot, as illustrated in Fig. 5 (c). A Fuzzy Logic Controller (FLC) has been integrated to expedite fault detection and active power control. It is noted that during a fault, the control system’s outer loop is disconnected due to the cessation of reactive power (Q) fluctuations, evidenced in Fig. 5 (a). Furthermore, all the above figures are merged into a single figure, as shown in Fig. 5 (d). Voltage stabilization in power grids is typically managed through reactive power control, which, while not directly beneficial, can enhance active power transmission capabilities and minimize active power losses when effectively regulated.

**Fig. 6:**
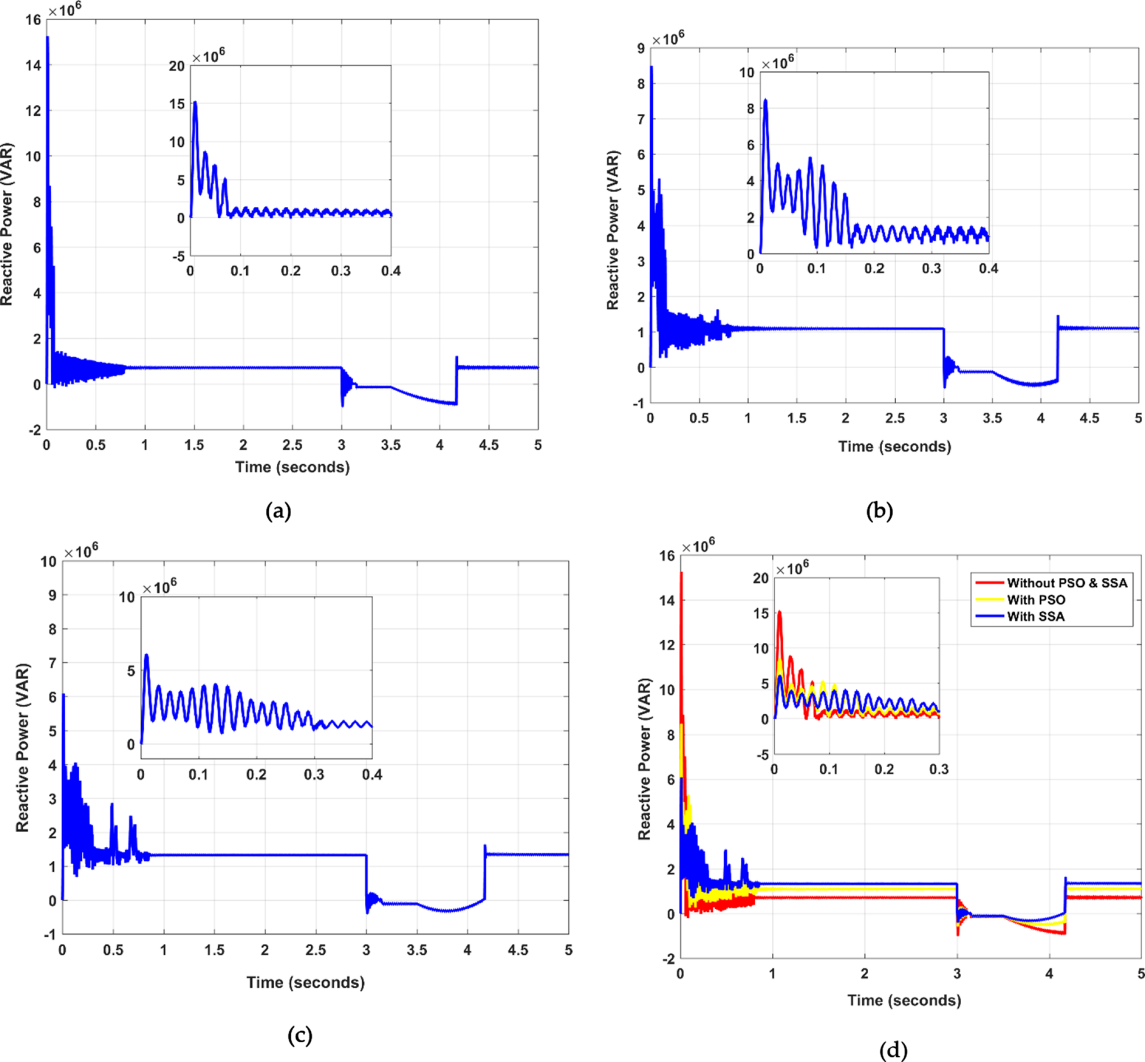
Reactive power (Q) analysis over a given time, (**a**) without PSO and SSA, (**b**) with PSO, (**c**) proposed with SSA, and (**d**) merged figures.

Distributed energy resources (DERs) are progressively entrusted with voltage stabilization roles by providing reactive power. The Rotor Side Converter (RSC) plays a dual role: optimizing power capture and managing active/reactive power. In the face of faults, DFIG should predominantly furnish rather than consume reactive power. Figure 6(a) illustrates the reactive power dynamics without applying optimization strategies. Performance attributes such as rise time, overshoot, and settling time for active/reactive power are analyzed through MATLAB simulations, with findings summarized in Table 5. As shown in Fig. 6 (b), the proposed model exhibits decreased overshoot in reactive power and diminished oscillations. As Fig. 6 (c) shows, utilizing the SSA optimization facilitates effective reactive power injection to the grid while concurrently reducing the DFIG’s reactive power output toward zero. Moreover, the SSA-optimized DFIG reduced the reactive power overshoot by 35% and settling time by 0.27 s compared to the PSO method. Furthermore, all the above figures are merged into a single figure, as shown in Fig. 6 (d).

**Table 5:** Dynamic response evaluation and analysis of active and reactive power curves.

Active power (*P*)			
Time (ms)	Without PSO, SSA	With PSO	With SAA
Initial / Overshoot	10.12 × 10^6	4.98 × 10^6	3.78 × 10^6
Rise time	7.396	6.995	5.514
Overshoot	24.339	19.313	21.442
Settling time	0.98	0.84	0.71
Reactive Power (Q)			
Initial / Overshoot	15.01 × 10^6	8.34 × 10^6	6.10 × 10^6
Rise time	2.644	4.084	2.344
Overshoot	90.426	56.296	48.783
Settling time	0.94	0.80	0.67

### Stator current (Is) and rotor current (ir) relationship

**Fig. 7:**
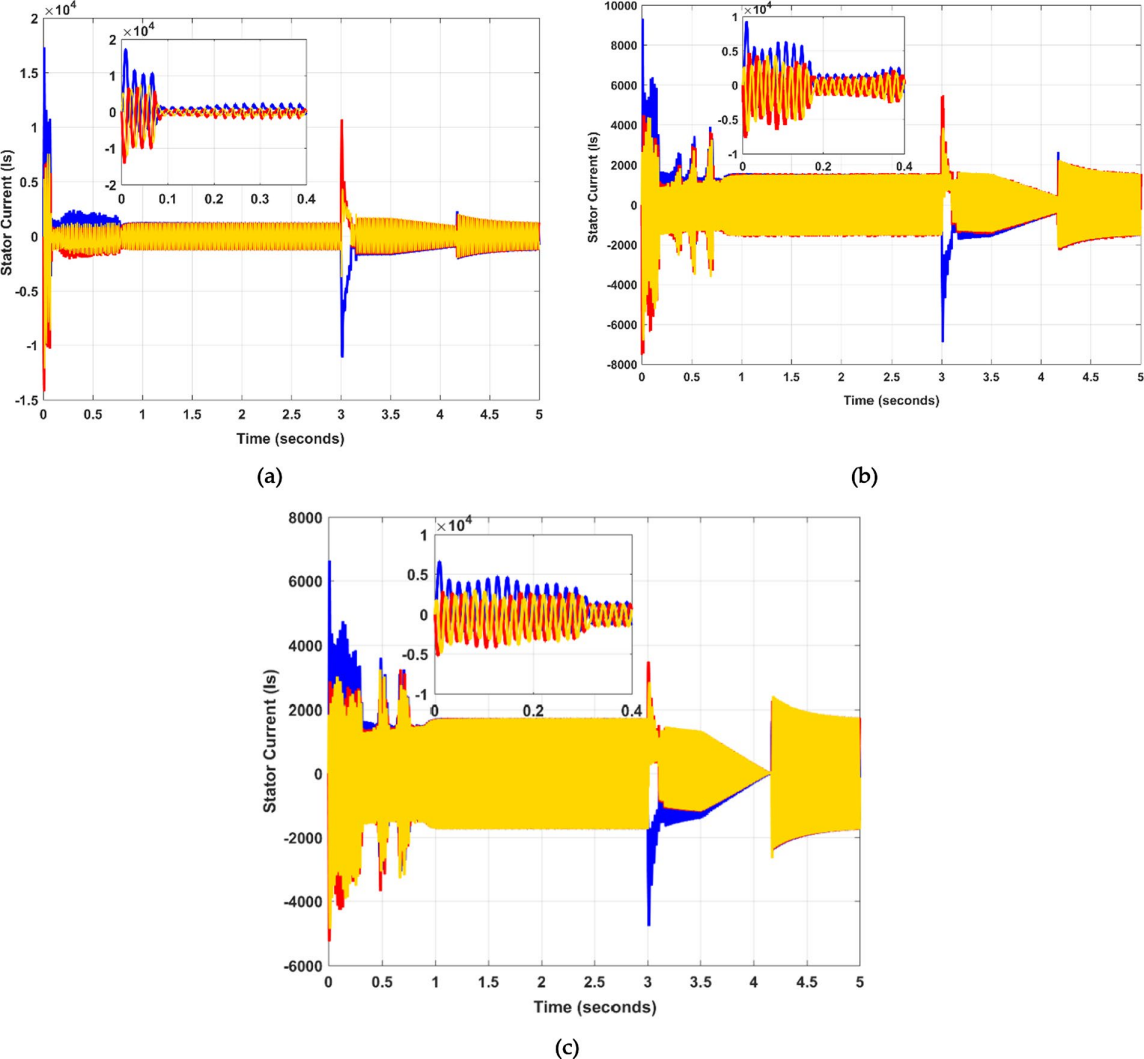
Stator Current (Is) representation of the proposed system (**a**) without SSA and PSO, (**b**) with PSO, and (**c**) proposed with SSA over a specified time.

The study indicates that while the applied techniques decrease the stator’s overshoot, they do not compromise the system’s connectivity. However, using the PSO algorithm, the overall overshoot is less, as shown in Fig. 7 (b). Harmonics in the power winding current are mitigated during the transient phase by employing the proposed SSA optimization strategy, as shown in Fig. 7 (c).

**Fig. 8:**
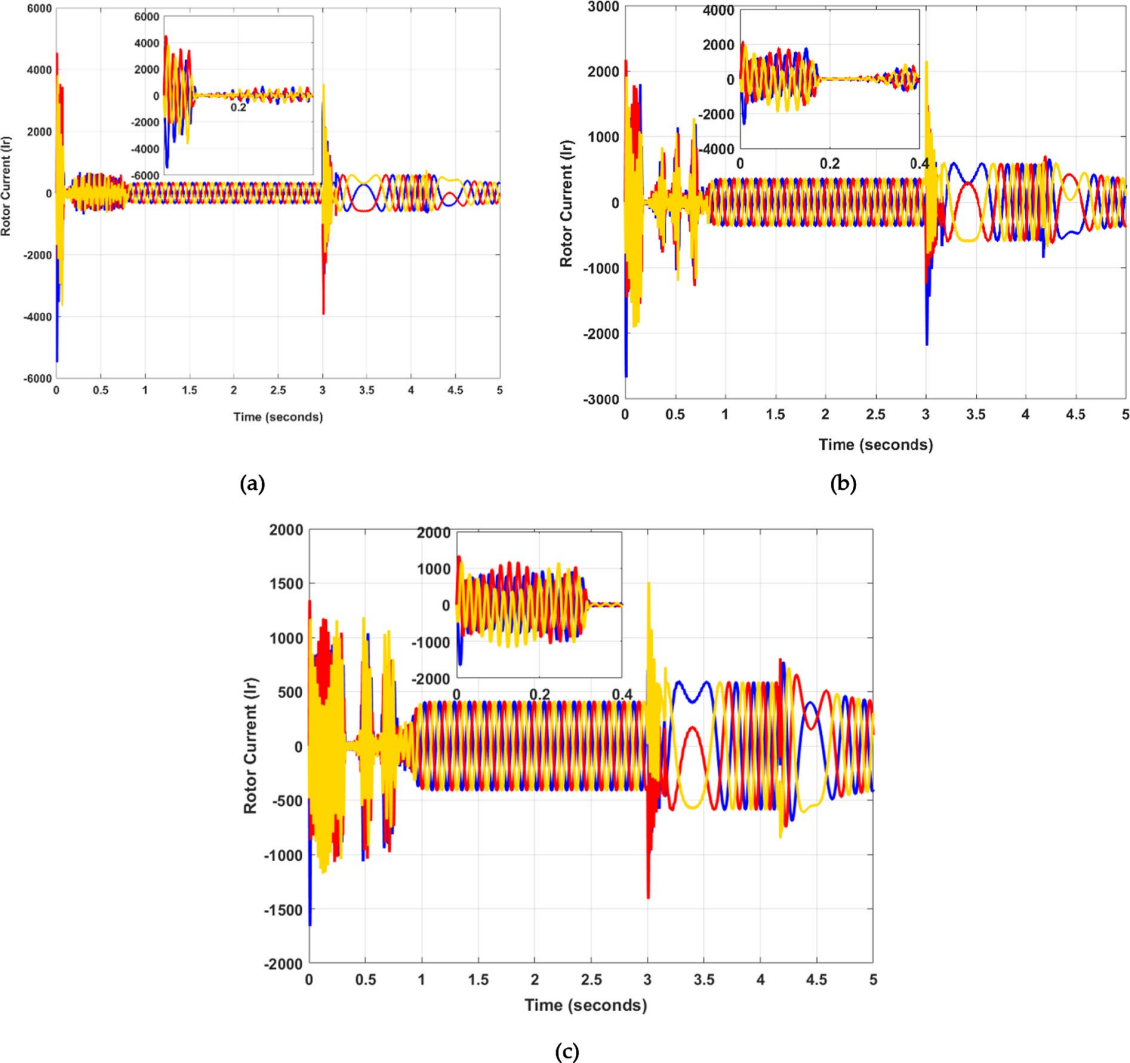
Rotor Current (Ir) illustration across the given time, (**a**) Without PSO and SSA, (**b**) with PSO, and (**c**) proposed with SSA.

 However, DFIG is an asynchronous machine that operates so that the rotor’s rotational speed is not synchronized with the grid frequency. It is understood that the operational characteristics of DFIG can be achieved by precise control of the rotor current. The rotor current experiences a large overshoot or peak value during a fault condition, as illustrated in Fig. 7 (a). The current magnitude plays a pivotal role in defining the machine’s dynamics. The primary aim of the rotor current is to steer it according to a predefined trajectory. By adjusting the rotor current’s amplitude and phase angle, the generator’s torque and the generation of active/reactive power can be effectively regulated. Moreover, by using the PSO optimization algorithm, the results are optimal, and the overshoot is also less, as shown in Fig. 8 (b). The overshoot or peak value is large during the fault period, as shown in Fig. 8 (a). In this paper, the SSA optimization algorithm has improved the machine dynamics. The proposed model demonstrates that during the fault occurrence at 3 s, the overshoot remains minimal, which is evident in Fig. 8c). The initial time, rise time, overshoot time, and settling time results of $\:{I}_{s}$ and $\:{I}_{r}$ calculated from MATLAB are shown in Table 6.

**Table 6:** Dynamic response evaluation of Stator and Rotor current curve.

Stator Current (Is)			
Time (ms)	Without PSO, SSA	With PSO	With SAA
Initial / Overshoot	16,500	8850	6200
Rising	11.812	8.465	3.005
Overshoot	19.313	13.770	8.939
Settling	0.82	0.79	0.75
Rotor Current (Ir)			
Initial / Overshoot	4110	2080	1255
Rising	20.798	20.723	1.785
Overshoot	141.347	67.723	25.501
Settling	0.80	0.78	0.73

### DC link voltage (vdc)

**Fig. 9:**
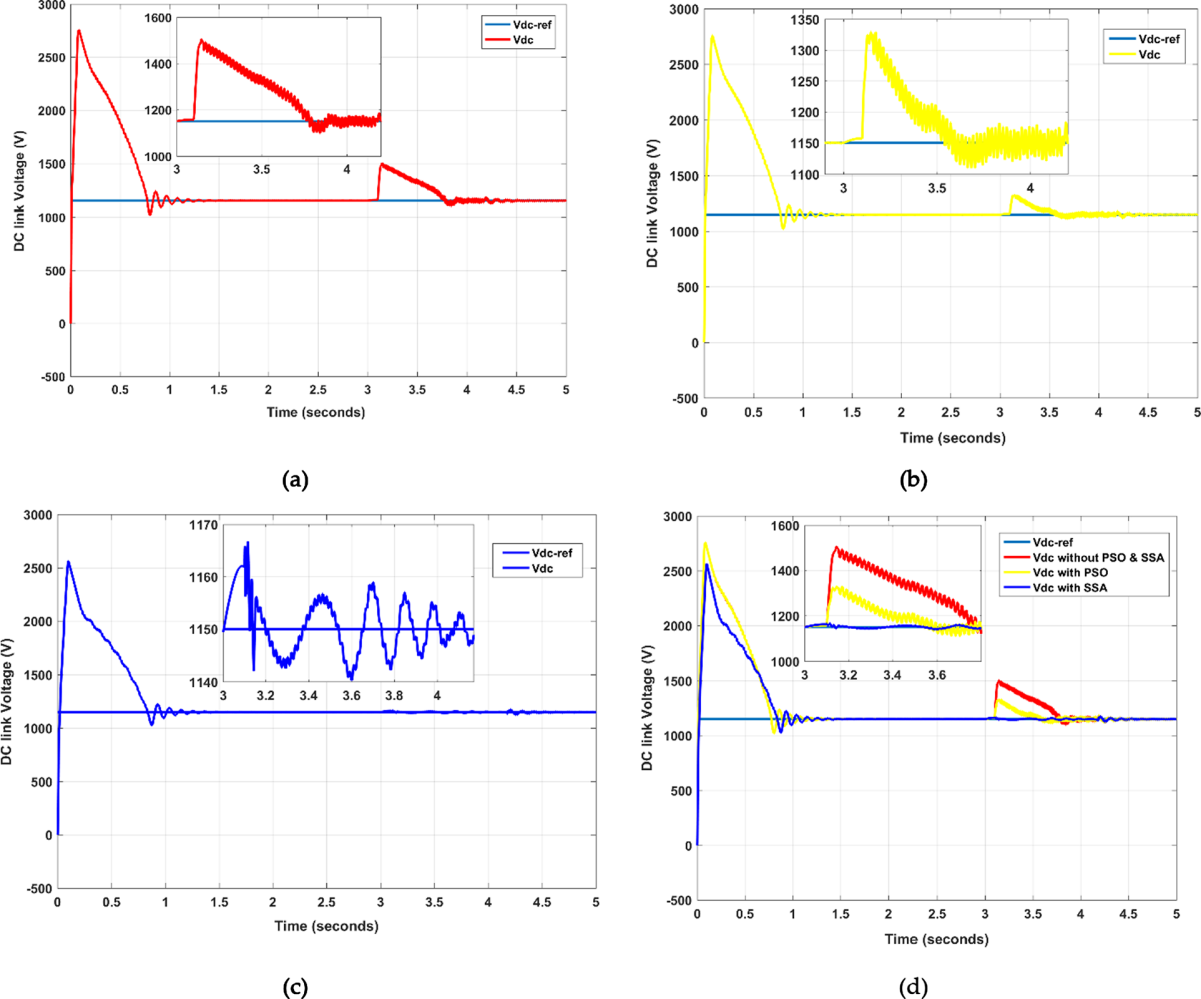
DC link voltage (Vdc) illustration across the given time, (**a**) Without PSO and SSA, (**b**) with PSO. (**c**) With SSA. (**d**) merged figures.

The Vdc is an intermediate stage in a power conversion system, which consists of a capacitor that stores the charge in the form of an electric field and smooths out the DC voltage. It acts as a backbone of energy conversion in power electronic systems to maintain the stability, reliability, and efficiency of the electrical systems. It is shown in Fig. 9 (a) that the Vdc results from 3 to 4 values without PSO and SSA algorithm as more ripple appears during this time period. However, by implementing the PSO algorithm, the results are somehow good, but there is still a ripple from 3 to 4 values, as sketched in Fig. 9 (b).

Moreover, it is improved by implementing the SSA algorithm in Fig. 9 (c), which shows that the ripple is improved to zero. Hence, better results are achieved during the time period 3 to 4. Furthermore, all the above figures are merged into a single figure, as shown in Fig. 9 (d).

### Discussion on the comparative analysis

The comparative analysis between the proposed technique and other state-of-the-art methods based on the quantified results provided below:

#### Fault ride-through (FRT) capability

The proposed SSA optimized the low-voltage ride-through (LVRT) capability more effectively than PSO and conventional methods. The SSA improved stability and reduced transient disturbances during fault conditions.

#### Dynamic response

The proposed algorithm improved the dynamic response of the DFIG system, resulting in a faster and more stable recovery from disturbances.

#### Quantitative results

- Active power (P): The initial overshoot with SSA was significantly lower, i.e., 3.78 × 10^6, as compared to 10.12 × 10^6 without optimization and 4.98 × 10^6 with PSO.
- Reactive power (Q): The overshoot in reactive power was reduced by 35% using SSA compared to PSO, and the settling time was improved from 0.94 ms to 0.67 ms.
- Stator and rotor Currents: The proposed method significantly reduced overshoot and improved settling times in both stator and rotor currents.

#### Control of active/reactive power

The FLC integrated with the SSA framework resulted in swift and accurate fault detection, which assists in the quicker activation of the compensation mechanisms during faults to maintain the power quality.

#### Comparative advantages

The proposed algorithm enhanced the operational parameters and showed a better performance in limiting fault current and managing the voltage dips effectively compared to PSO and conventional techniques. Henceforth, the comparative analysis emphasizes the efficiency of the proposed SSA and FLC approach in refining machine dynamics and improving fault management capabilities of DFIG systems, which means presenting a robust solution to improve the resilience and efficiency of wind energy systems.

Although various optimization techniques are available in the literature review, each suffers from inherent limitations, which motivates the author to search for a better search mechanism and converge to even the best solution. The proposed Salp swarm optimization techniques consist of three main equations: exploration, exploitation, and balancer equation. Unlike the existing state-of-the-art algorithms, the proposed SSA avoids a biased search mechanism where the particles follow the best particle; in case the assumed best particle is locally optimal, then all the particles follow the unguided path. Unlike those mechanisms, the proposed SSA follows a random search mechanism in a bounded path, avoiding a biased search mechanism. Secondly, the SSA balancer parameter c1 employs an exponential search rather than a linear search mechanism. The linear search mechanism (just like PSO employs, widely adopted in literature) has a higher diversity of particles in search space, which results in missing the optimal value; however, the SSA employs an exponential search mechanism, which results in a higher diversity of particles in exploration part and lower diversity of particles in exploitation part, resulting in appropriate searching. The results are evident from Table 5 in the results section.

## Conclusion

In the present study, a new approach has been proposed to improve the low voltage ride-through (LVRT) proficiency of a doubly fed induction generator (DFIG) by controlling the reactive current through its rotor side converter (RSC). This strategy aims to lighten voltage drops during short-circuit disturbances. The employed model incorporates a DFIG with a corresponding equivalent circuit, applying a single-phase representation of the RSC to assess its steady-state influence on voltage reductions. A fuzzy logic controller (FLC) was developed to swiftly detect LVRT conditions when faults occur, using the power windings’ frequency and voltage magnitude as inputs. The results obtained from the dynamic response evaluation of the stator and rotor current Curve show that the initial time, rise time, overshoot time, and settling time Stator Current and Rotors were found to be 6200 ms, 3.005 ms, 8.939 ms, 0.75 ms and 1255 ms, 1.785 ms, 25.501 ms, 0.73 ms with SSA which are highly improved as compared to the results given by the PSO and without PSO, SSA together respectively. It is evident from simulation results that the overshoot of active power is being improved, which is decreasing from 10.12 × 10^6 to 3.78 × 10^6 with the help of the SSA algorithm. Moreover, the best results are also achieved with reactive power, which improves and decreases from 15.01 × 10^6 to 6.10 × 10^6. Validations through MATLAB / Simulink simulations demonstrated the proposed controller’s efficacy, significantly reducing overshoot duration and transient disturbances. It is essential to note that the approach primarily addresses the steady-state current to counter voltage sags during faults. Incorporating the transient current behavior could further refine performance and dampen fault-induced oscillations. In addition, while the FLC enhances the RSC’s response to disturbances, its success is contingent upon the precise formulation and adjustment of the rules in its knowledge base, underlining the importance of meticulous design and calibration for optimal functionality.

## Future work

Future work based on the proposed study could explore integrating transient current behavior into DFIG models to improve fault response and lessen oscillations. More state-of-the-art hybrid control strategies integrating fuzzy logic with other optimization algorithms, i.e., Genetic Algorithms or Deep Learning, could be established for robust fault management. The scope of the future study could be extended by including real-time testing and the integration of DFIGs with other RES and storage systems, as well as a modified demagnetizing control strategy, which could validate the proposed strategies’ efficiency in diverse operational arenas. Moreover, the comparative analysis with the latest optimization algorithms would categorize the optimal solutions for improving the fault ride-through capabilities of DFIG systems in wind energy conversion.

## Data Availability

Data will be made available on request to Corresponding Author.

## Abbreviations

DFIG: Doubly-fed Induction Generator
RSC: Rotor Side Converter
GSC: Grid Side Converter
WECS: Wind Energy Conversion System
LVRT: Low Voltage Ride Through
FLC: Fuzzy Logic Control
FRT: Fault Ride Through
SSA: Salp Swarm Algorithm
MPPT: Maximum Power point Tracking
DERs: Distributed Energy Resources
PSO: Particle Swarm Algorithm
ITF: Interturn Fault
DG: Distributed Generation
PMSG: Permanent Magnet Synchronous Generator
BDFIG: Brushless Doubly-fed Induction Generator
MPC: Model Predictive Control
GA: Genetic Algorithm
GWO: Gray Wolf Optimizer
PI: Proportional Integral
MATLAB: Matrix Laboratory
ZO: Zero
PS: Positive Small
PM: Positive Medium
PB: Positive Big
